# Supplementary material for: Ensuring confidentiality and privacy of cloud data using a non-deterministic cryptographic scheme
Source: PLoS One. 2023 Feb 9;18(2):e0274628. doi: 10.1371/journal.pone.0274628 (PMC9910760; doi:10.1371/journal.pone.0274628)
Supplement: Attachment — Submitted filename: Plos one reviewers comment.docx [file pone.0274628.s002.docx]

Dear Editor,

Thanks very much for the time given for the revision of my manuscript. Much effort has been put into revising the manuscript to meet your standard.

Below are a point-by-point rebuttal to the reviewers’ comments.

All corrections have been marked red.

Hoping to hear from you soon.

Many thanks.

REVIEWER #1

| COMMENT | REBUTTAL/ CORRECTION | PAGE(S) |
| --- | --- | --- |
| Improve the quality of provided figures. | The quality of figures has been improved using Edraw tools | 2,3, 7, 10, 11, 15, 16 |
| Add formal security analysis using the RoR model | Much effort has been put to implement the proposed algorithm using RoR model but not very responding |  |
| The threat model should be in its proper form. | This has been well implemented in the manuscript | 7 |
| Proposed scheme should be compared with some recently published schemes. | The proposed model is compared with state-of-the-art algorithms and their execution times are compared. | 16 |
| Comparisons of communication cost, computation cost, storage cost, and "security and functionality features" are not available | The computational cost, security, and functionality features are illustrated but the storage cost and throughput are considered to be implemented in a comparison paper for an already published paper with inderscience dubbed” An enhanced RSA algorithm using Gaussian interpolation formula” | 15 |

REVIEWER # 2

| COMMENT | REBUTTAL/ CORRECTION | PAGE(S) |
| --- | --- | --- |
| There are many grammatical errors in the manuscript. So strong English proof reading is required. For example, “content is kept as secured as possible [16]” must be “content is kept as secure as possible [16]”. Also, check the statement “This helped to quicken the convolution and also helps in the analysis” | An English grammar correction tool has been used to correct all grammatical errors in the manuscript. | 3 |
| In page 14, what is the meaning of “Using Good Prime generate two prime numbers P and Q”. Also before this step, the authors should have used Select and select Q step which is used as the last step in Figure 4. | This has been corrected in the manuscript | 7 |
| There are many formatting mistakes in the paper. For example, in page 15, after equation (2) a citation [27] has been used which should be given inside the text. | The formatting has now been improved | 8 |
| In the results and analysis section, the authors should do mathematical analysis between the proposed work with existing schemes. So table 3 and 4 should be modified. | These corrections have been modified as now shown in tables 5 and 6 | 16 |
| Security analysis must be done using some formal verification tools or formal mathematical analysis. | Security analysis was done given the snippet of the output shown in the manuscript. | 12, 13 |
| Related work section is weak. Because Some important recent references are missing, the following references must be totally added in the Section "References" (otherwise, the reference is not enough, then it must be revised again until it is enough): | The related work has been updated using the provided papers. | 6 |
